# Optimized Ammonia-Sensing Electrode with CeO_2_/rGO Nano-Composite Coating Synthesized by Focused Laser Ablation in Liquid

**DOI:** 10.3390/nano14151238

**Published:** 2024-07-23

**Authors:** Mengqi Shi, Hiroyuki Wada

**Affiliations:** School of Materials and Chemical Technology, Tokyo Institute of Technology, 4259 Nagatsuta-cho, Midori-ku, Yokohama 226-8502, Japan; shi.m.aa@m.titech.ac.jp

**Keywords:** nanoparticle, CeO_2_/rGO composite, laser ablation in liquid, electrochemical sensing, ammonia detection, synergistic effects

## Abstract

This study investigated the synthesis of cerium oxide (CeO_2_) nanoparticles (NPs) and composites with reduced graphene oxide (rGO) for the enhanced electrochemical sensing of ammonia. CeO_2_ NPs were prepared by the focused laser ablation in liquid (LAL) method, which enabled the production of high-purity, spherical nanoparticles with a uniform dispersion and sizes under 50 nm in a short time. The effects of varying irradiation fluence and time on the nanoparticle size, production yield, and dispersion were systematically studied. The synthesized CeO_2_ NPs were doped with rGO to form CeO_2_/rGO composites, which were drop casted to modify the glassy carbon electrodes (GCE). The CeO_2_/rGO-GCE electrodes exhibited superior electrochemical properties compared with single-component electrodes, which demonstrated the significant potential for ammonia detection, especially at a 4 J/cm^2^ fluence. The CeO_2_/rGO composites showed uniformly dispersed CeO_2_ NPs between the rGO sheets, which enhanced the conductivity, as confirmed by SEM, EDS mapping, and XRD analysis. Cyclic voltammetry data demonstrated superior electrochemical activity of the CeO_2_/rGO composite electrodes, with the 2rGO/1CeO_2_ ratio showing the highest current response and sensitivity. The CV response to varying ammonia concentrations exhibited a linear relationship, indicating the electrode’s capability for accurate quantification. These findings highlight the effectiveness of focused laser ablation in enhancing nanoparticle synthesis and the promising synergistic effects of CeO_2_ and rGO in developing high-performance electrochemical sensors.

## 1. Introduction

Monitoring ammonia (NH_4_⁺) levels in the biomedical field is critically important due to its direct implications on human health [1,2], particularly in patients with liver disease [3]. The liver processes ammonia by converting it into urea for excretion [4]. However, in conditions such as cirrhosis [5], the liver’s capacity to handle ammonia diminishes, leading to its accumulation in the blood. Elevated ammonia levels can precipitate hepatic encephalopathy [6], which is a serious neuropsychiatric syndrome characterized by cognitive dysfunction, altered consciousness [7], and potentially a coma [8]. The necessity of effective ammonia monitoring is underscored by the metabolic origins of ammonia in the body, which is primarily produced from the catabolism [9] of amino acids in various organs, including the intestines [10], muscles [11], and kidneys [12]. Given the potential for ammonia to cause severe health complications, the real-time monitoring of blood NH_4_⁺ levels is essential [13], making ammonia sensors vital tools in managing liver diseases and preventing the onset of hepatic encephalopathy. Additionally, integrating these modified carbon-based sensors into portable devices for real-time monitoring or their use in clinical settings [14] for patient diagnostics could significantly enhance patient care and disease management.

Electrochemical sensors are highly effective for detecting and monitoring gases due to their high sensitivity, selectivity, and rapid response times [15,16,17]. These sensors work by generating electrical signals when target molecules interact with electrodes [18]. In this research, a CeO_2_/rGO glassy carbon electrode (GCE) was designed to detect ammonia. The CeO_2_/rGO composite electrode combines cerium oxide nanoparticles with reduced graphene oxide (rGO) for a synergistic effect [19] to enhance the sensor’s performance. CeO_2_ provides catalytic activity [20], while rGO offers high conductivity [21] and a large surface area [22] for electron transfer. The synergistic effect between CeO_2_ and rGO significantly improves the electrochemical activity of the electrode, making it highly sensitive and effective for ammonia detection. To fully exploit the synergistic properties, the uniform spherical size and high dispersion of CeO_2_ nanoparticles are crucial to increase the specific surface area and electron transport of the reaction [23,24].

To increase the production and improve the quality of CeO_2_ nanoparticles for sensor applications, this study employed a focused laser ablation in liquid (LAL) method [25]. Compared with our previous research [26], this technique aims to produce smaller, uniformly dispersed, and spherical CeO_2_ nanoparticles with enhanced production efficiency [27] and less aggregation [28]. The focused laser beam intensifies the energy density at the target site, promoting more efficient ablation and higher nanoparticle yield [29]. This method allows for producing high-purity CeO_2_ nanoparticles in a very short time, significantly boosting the overall production efficiency [30]. When the focused laser beam irradiates the target material, the atoms absorb the laser energy, causing rapid thermal expansion and evaporation, leading to the ejection of surface atoms [31,32]. These ejected atoms and clusters then rapidly cool and condense in the surrounding liquid medium, forming nanoparticles [33]. The rapid cooling stabilizes the nanoparticles at the desired size and shape, preventing further agglomeration [34]. Compared with other synthesis methods, LAL is a green physical technique [35] that offers controllable parameters [36] and the capability to achieve high temperatures without generating by-products [37]. This method ensures a high yield and uniformity of the resulting nanoparticles, enhancing their suitability for sensor applications by providing consistent and high-quality coatings.

In this research, by optimizing the preparation method of CeO_2_ nanoparticles through the focused laser ablation method, we achieved high-purity nanoparticles with uniform dispersion in a very short time and with a high production efficiency. The mechanisms of irradiation fluence and the time effect on the nanoparticle size, production yield, and uniform dispersion through this sintering process were investigated. Our results demonstrated that the optimized CeO_2_ nanoparticles synthesized via focused laser irradiation exhibited a high purity, a spherical shape, and uniform dispersion of a small size, which is essential for effective sensor applications. Furthermore, the CeO_2_/rGO composite electrodes were shown to have superior electrochemical properties compared with single-component electrodes, highlighting their significant potential for ammonia monitoring and broader electrochemical sensing applications. This study underscored the effectiveness of focused laser ablation in enhancing nanoparticle synthesis and the promising synergistic effects of CeO_2_ and rGO in developing high-performance electrochemical sensors.

## 2. Experimental Methods

The CeO_2_ targets were first prepared under a 100 MPa pressure by a tablet machine. This was followed by a sintering process: 900 °C for 2 h and 1250 °C for another 2 h, resulting in tablets with an approximate thickness of 0.3 mm after sintering shrinkage. The sintered CeO_2_ target was placed in a glass bottle with 5 mL of pure water. An Nd:YAG laser (532 nm, 10 Hz, 13 ns pulse width) was used for ablation after the adjustment of PBS and HWP, and the irradiation beam was focused by a lens onto the target with fluences of 2, 4, 6, and 8 J/cm^2^ for various durations (10, 20, 30, 40, 50, and 60 min).

rGO was prepared by sintering graphene oxide at 500 °C, followed by ultrasonication. A total of 8 g of rGO was dispersed in 50 mL of pure water and ultrasonicated for 2 h according to our previous study [38]. CeO_2_ NPs were added to the rGO suspension in molar ratios of 4:1, 2:1, 1:1, 1:2, and 1:4, and were dispersed by ultrasonication and stirring for 30 min. After 24 h of precipitation, the mixture was centrifuged and freeze dried. The resulting CeO_2_-rGO composites were drop casted onto glassy carbon electrodes (GCEs), applied five times, and oven dried at 80 °C.

The morphology of CeO_2_ nanoparticles synthesized via the focused LAL process was characterized using SEM (Hitachi High-Tech. Co. S-4800, Tokyo, Japan) and DLS (Sysmex Co. Zetasizer Nano, Hyogo, Japan). UV–Vis spectroscopy (Jasco. Co. V-670, Tokyo, Japan) was employed for the production calculation of nanoparticles. Both the raw materials and synthesized CeO_2_ nanoparticles were identified using XRD (Philips PANalytical, X’Pert-Pro-MRD, Almelo, The Netherlands). Energy-dispersive X-ray spectroscopy (EDS, 200 kV, JEM-2010F, Co. JEOL, Tokyo, Japan) was used to analyze the elemental composition of the CeO_2_ nanoparticles. Cyclic voltammetry (CV, RRDE-3A Rotating Ring Disk Electrode Apparatus Ver.3.0, Co. BAS, Numazu, Japan) was used to evaluate the electrochemical properties and sensing performance of the synthesized CeO_2_-rGO nanocomposites.

## 3. Results and Discussion

**Figure 1:**
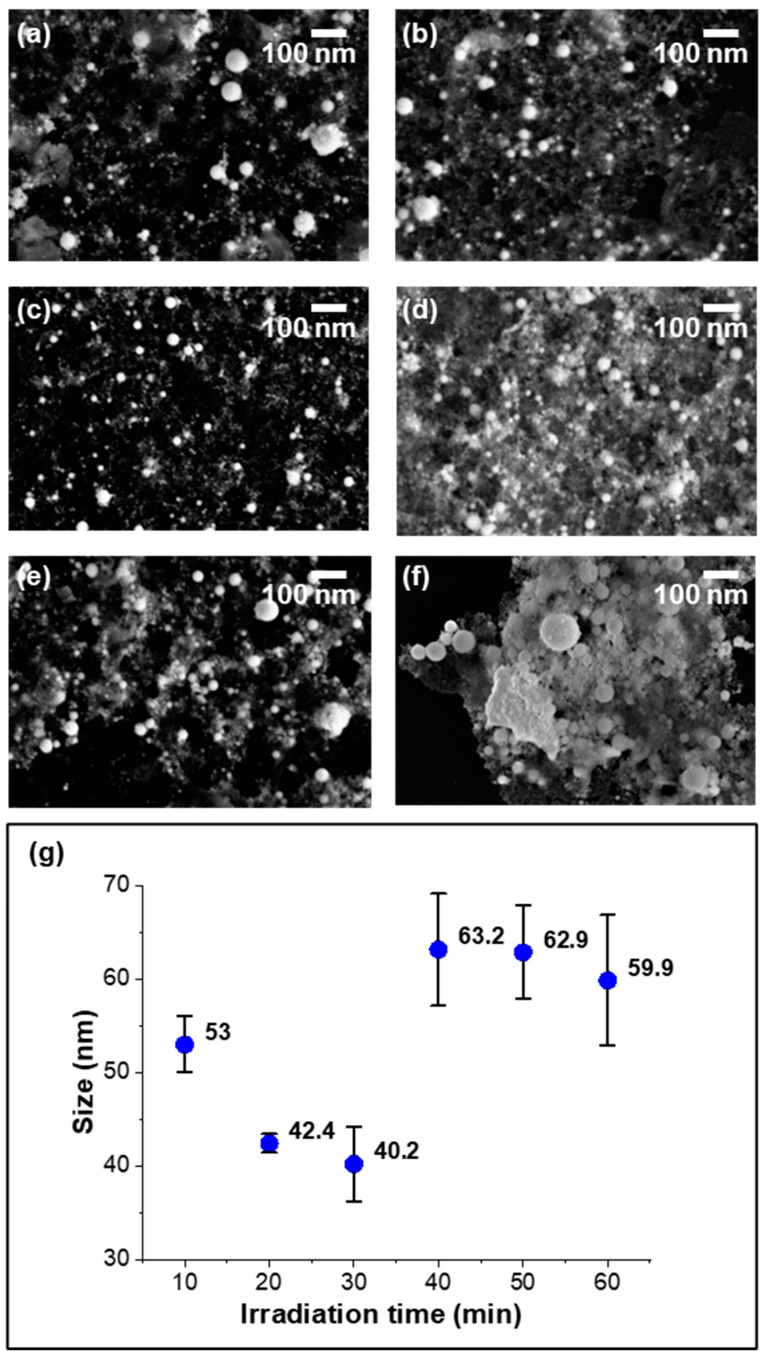
SEM morphology of CeO_2_ nanoparticles ablated under a 5 J/cm^2^ fluence, with irradiation times of (**a**–**f**) 10, 20, 30, 40, 50, and 60 min, and (**g**) a histogram of average particle size with increased time.

Figure 1 illustrates the size variation of the CeO_2_ nanoparticles synthesized via laser ablation in liquid (LAL) under a fluence of 4 J/cm^2^ and affected by increased irradiation time. This method effectively produces smaller and more uniformly sized nanoparticles compared with our previous research of non-beam-concentration techniques on suspension liquids [26]. The SEM figures show the existence of CeO_2_ nanoparticles in spherical shapes for each time duration, even for 10 min. The production of the nanoparticles also increased with prolonged irradiation time. This rapid formation process formed a plasma plume that interacted with the liquid, which peeled and cooled the nanoparticles [39]. Simultaneously, the CeO_2_ nanoparticles repeatedly underwent processes of melting, re-melting, and re-explosion, which resulted in a dynamic environment that favored the continuous production of small, uniformly sized particles. As the irradiation time increased, the SEM images show a gradual change in the nanoparticle morphology. Initially, the average particle size decreased with increased irradiation time and reached a minimum of around 30 min, indicating an efficient ablation process over time. The size distribution (Figure 1g) shows that at this stage, the primary particle sizes of the CeO_2_ nanoparticles decreased with the irradiation fluence increase. However, over 30 min, the primary particle size began to increase. This increase was likely due to the higher nanoparticle concentration in the liquid, where particles obstructed the laser path, which resulted in an insufficient irradiation fluence for some particles.

**Figure 2:**
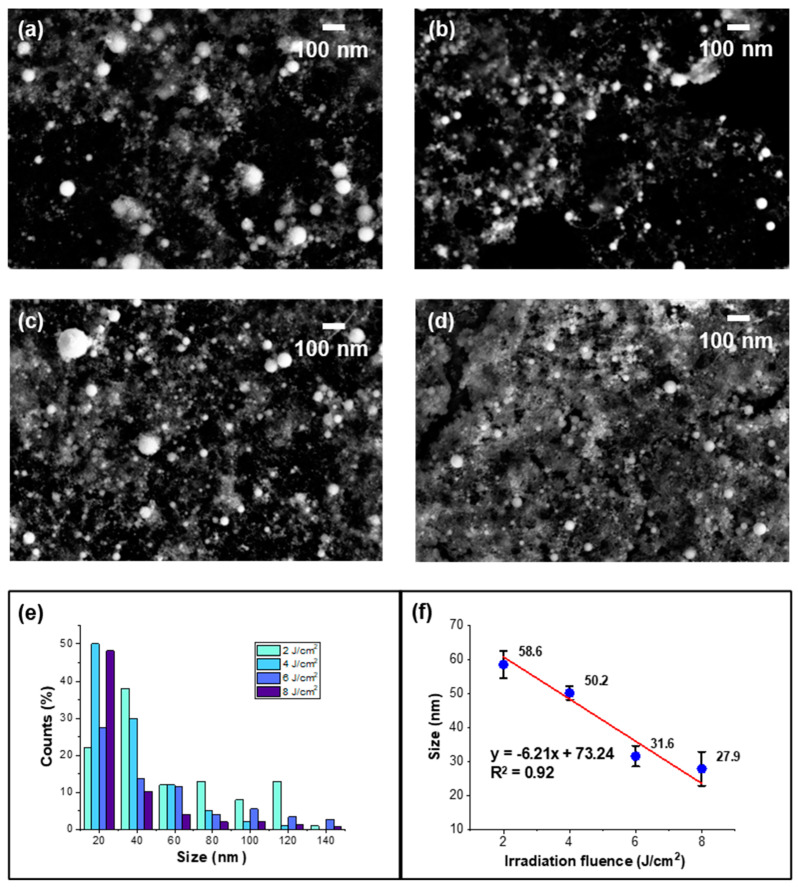
SEM morphology of CeO_2_ nanoparticles ablated with an irradiation time of 30 min under irradiation fluences of (**a**–**d**) 2, 4, 6, and 8 J/cm^2^. (**e**) The primary size distribution and (**f**) average size of nanoparticles as a function of the laser fluence with a linear fitting.

To study the irradiation fluence effect on the CeO_2_ formation process, Figure 2 shows the SEM morphology of the CeO_2_ nanoparticles synthesized under the irradiation fluences of 2, 4, 6, and 8 J/cm^2^. The SEM images (a–d) show that as the irradiation fluence increased, the primary particle size decreased, as evidenced by the histogram of size distribution in Figure 2e and the average primary particle size (f). The linear fitting between the fluence and particle size indicates an inverse relationship, with a high R^2^ value of 0.92. This phenomenon could be attributed to the higher energy density provided by the increased fluence, which facilitated more efficient ablation and rapid cooling and led to the formation of smaller nanoparticles. At higher fluences, the energy delivered to the target material was sufficient to create a high-density plasma plume. This plasma plume enhanced the fragmentation of the target material into smaller nanoparticles.

However, with the irradiation fluence increase, particularly beyond 4 J/cm^2^, the SEM images (c, d) revealed a significant flocculent background beneath the CeO_2_ nanoparticles. This melting phenomenon occurred because of the laser-induced heating of the spinodal temperature in some areas, which was higher than the melting point of CeO_2_ [40]. Consequently, the material partially melted, which created a molten environment that facilitated the coalescence of nanoparticles into larger, irregular shapes. This effect was more pronounced at higher fluences due to the increased energy deposition and spinodal temperature, which not only ablated the target material but also induced localized melting. Also, due to the increased concentration by the effect of the higher fluence, clusters formed, as shown in Figure 2c,d. Therefore, we believe the irradiation fluence before the target melting (4 J/cm^2^) is the most suitable condition for preparing small-sized spherical CeO_2_ nanoparticles.

**Figure 3:**
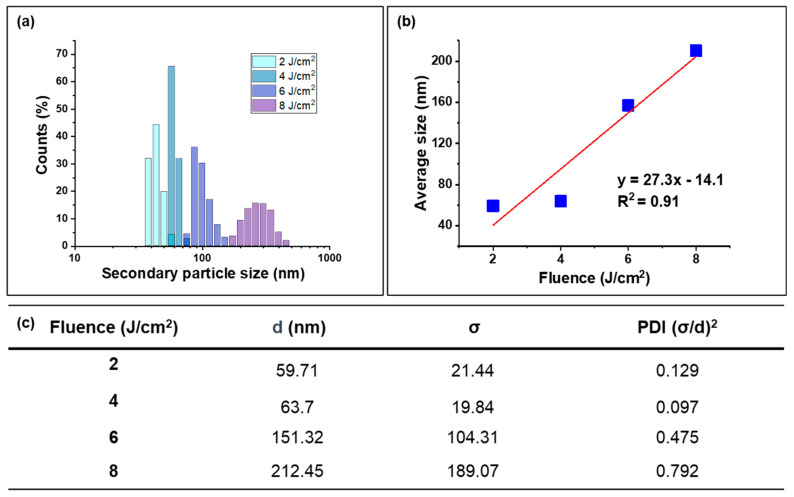
(**a**) DLS histogram of secondary particle size distribution with an irradiation time of 30 min under irradiation fluences of 2, 4, 6, and 8 J/cm^2^. (**b**) Average secondary particle size as a function of energy fluence with the linear fitting. (**c**) PDI calculation of nanoparticles under each fluence.

The secondary particle size distribution of CeO_2_ nanoparticles was measured by dynamic light scattering (DLS), as shown in Figure 3. The secondary particle size increased with increased fluence, and the linear fitting (Figure 2b) shows a positive relationship, which was in contrast with the primary particle size trend observed in the SEM data (Figure 2). This discrepancy could be attributed to the aggregation phenomenon, which was driven by several factors, such as the Van der Waals force [41], local electric charges [42], and the specific surface area [43] and led to minimizing the surface energy, which gave a thermodynamically stable status. As the fluence increased, more nanoparticles were generated, which led to higher particle concentrations in the solution. This higher concentration increased the likelihood of collisions and interactions between particles and resulted in the aggregation and formation of larger secondary particles. Additionally, as shown in the SEM image, the high-energy input at increased fluence levels could induce partial melting of the nanoparticles, which caused them to coalesce into larger aggregates upon cooling.

To analyze the size distribution of the secondary particles, the polydispersity index (PDI) was calculated by Formula (1):

(1) $$PDI={\left(\frac{\sigma }{d}\right)}^{2}$$

where *σ* is the standard deviation and *d* is the average particle size. The data in Figure 2c shows that the PDI increased significantly after aggregation and melting, indicating a broader and less uniform size distribution at higher fluences. Specifically, at a fluence below 4 J/cm^2^, the PDI value was around 0.1, suggesting a monodisperse system of CeO_2_ nanoparticles. However, as the fluence increased beyond 4 J/cm^2^, the PDI values rose, even to a PDI value of 0.792 under a fluence of 8 J/cm^2^, indicating an uneven size distribution of nanoparticles and reflecting the increased heterogeneity in particle size due to aggregation and melting effects.

**Figure 4:**
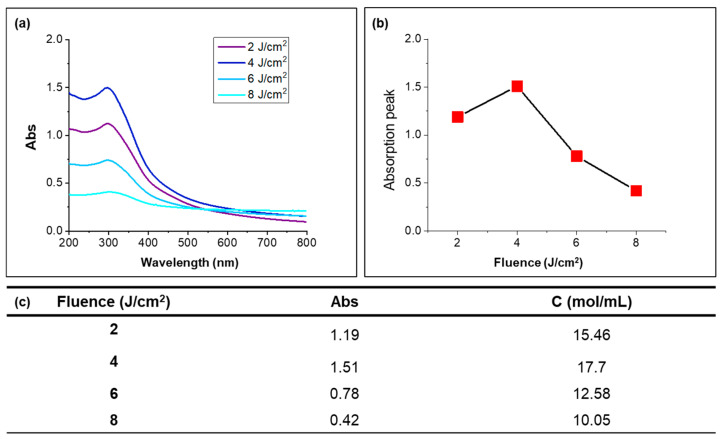
(**a**) UV–vis spectrum of CeO_2_ nanoparticles ablated under each fluence with an irradiation time of 30 min. (**b**) Absorption peak intensity as a function of the irradiation fluence. (**c**) Concentration calculation under each fluence.

The UV–visible data of the CeO_2_ nanoparticle suspension obtained after dilution 10 times were analyzed. Figure 4a shows the UV–vis spectra, where the suspension irradiated at 4 J/cm^2^ exhibited the highest absorption value, and this peak absorption was attributed to the large amount of dispersed nanosized CeO_2_ particles, which showed smaller and more uniformly sized nanoparticles at this fluence. As the fluence increased beyond 4 J/cm^2^, the absorption values tended to decrease, as depicted in Figure 4b, which can be explained by the aggregation phenomenon at higher fluences, which was consistent with the SEM (Figure 2) and DLS (Figure 3) data, where the agglomerated nanoparticles reduced the specific surface area for absorbing light and precipitate, which resulted in a decrease in the absorption peak.

The concentration of CeO_2_ nanoparticles was calculated using the Beer–Lambert law [44] (Formula (2)), which relates the absorbance (*A*) to the concentration (*C*), path length (*l*), and molar absorptivity (*ε*):

(2) A=εlC

Using the known concentration from the Supplementary Materials (Figure S1) and its corresponding UV–vis absorbance value, the molar absorptivity (ε) was determined, and the concentrations of CeO_2_ nanoparticle suspensions under different fluences are shown in Figure 4c. From the UV–vis data and subsequent calculations, the highest production of CeO_2_ nanoparticles was achieved at 17.7 mol/L and 4 J/cm^2^, which is approximately five times higher than the concentration produced using non-focused irradiation processes [25]. This significant increase in nanoparticle yield demonstrated the efficiency of the focused laser ablation method in generating high concentrations of well-dispersed CeO_2_.

**Figure 5:**
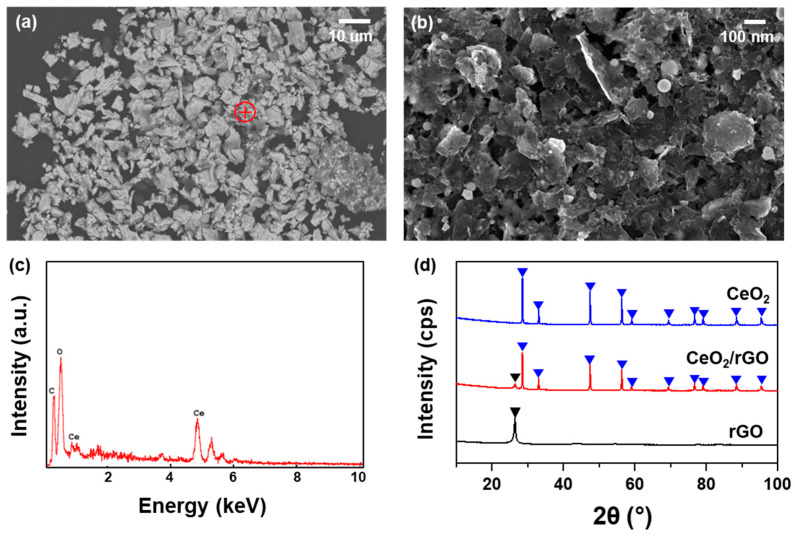
(**a**) SEM image of CeO_2_/rGO composite in a molar ratio of 1:1 at low magnification; (**b**) SEM image at high magnification of the red pointed position in (**a**); (**c**) EDS mapping of the element distribution at the red point in (**a**); (**d**) XRD pattern of the CeO_2_ nanoparticles, rGO, and CeO_2_/rGO composite.

The morphology of the CeO_2_/rGO composite in a molar ratio of 1:1 is shown in Figure 5 through various characterization techniques. The SEM images (Figure 5a,b) at different magnifications demonstrate the uniform dispersion and combination of CeO_2_ nanoparticles into the gaps between the reduced graphene oxide (rGO) sheets, indicating that the rGO sheets served as an excellent support material for the CeO_2_ nanoparticles and enhanced the synergistic effects of the composite. The EDS mapping (Figure 5c) confirmed the presence of carbon (C), oxygen (O), and cerium (Ce) elements within the composite, with no detectable impurities. This elemental uniformity further supports the successful integration of CeO_2_ nanoparticles into the rGO matrix. The XRD patterns (Figure 5d) show distinct peaks corresponding to the (111), (200), (220), (311), (222), (400), (331), (420), and (422) crystallographic planes, which are characteristic of the cubic fluorite structure of CeO_2_. Additionally, the rGO pattern exhibited the (002) peak, which was significantly reduced after ultrasonication due to the adsorption of CeO_2_ nanoparticles between the rGO sheets. This adsorption reduced the interlayer spacing of rGO, and also because of the attenuation effect of CeO_2_ on X-rays, this led to a marked decrease in the intensity of the rGO diffraction peaks. The absence of any additional peaks in the XRD pattern indicates that there were no second phases present in the nanocomposites, suggesting a high purity of the CeO_2_/rGO composite.

**Figure 6:**
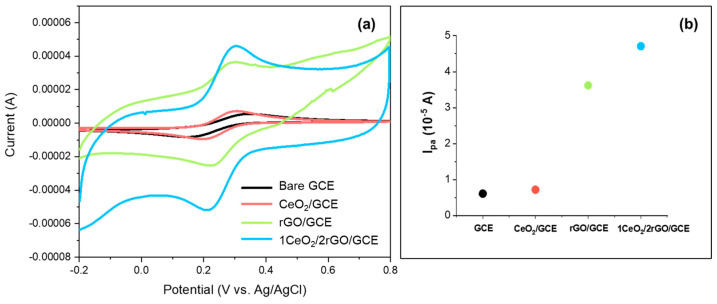
(**a**) Cyclic voltammetry plots of GCE with different coatings (bare GCE, CeO_2_/GCE, rGO/GCE, 1CeO_2_/2rGO/GCE) in the presence of 1 mM K_3_[Fe(CN)_6_] in 0.5 M KCl, with a scan rate of 10 mV/s. (**b**) The anodic peak current of each coating.

Cyclic voltammetry (CV) was used to measure the conductivity and sensitivity of the composite as an electrode coating for ammonia. The working electrode area was 0.07 cm^2^, with a sample interval of 5 mV under a 25 °C electrolyte temperature. Figure 6 shows the CV plot for different GCE coatings—bare GCE, CeO_2_/GCE, rGO/GCE, and CeO_2_/rGO/GCE (CeO_2_ and rGO in a molar ratio of 1:2)—in a 0.5 M KCl solution with 1 mM K_3_[Fe(CN)_6_] at a scan rate of 10 mV/s. The bare GCE showed a negligible current response, indicating poor electrochemical activity. The CeO_2_/GCE slightly improved the current response of the anodic peak (I_pa_ = 0.74 × 10^−5^ A), while rGO/GCE showed a significantly higher anodic peak (I_pa_ = 3.62 × 10^−5^ A) due to the rGO’s high conductivity and surface area. The 1CeO_2_/2rGO/GCE demonstrated the highest electron transfer and current response for the anodic peak (I_pa_ = 6.67 × 10^−5^ A), indicating that the combination of CeO_2_ and rGO enhanced the oxidation reaction. This enhancement was due to the synergistic effect between rGO and CeO_2_ nanoparticles like in a p–n heterojunction structure, where the oxygen-functional groups on the GO could facilitate the transfer of electrons as electron acceptors, and the CeO_2_ could donate or accept oxygen vacancies and electrons. This enhanced current response indicates that the CeO_2_/rGO composite is a promising candidate for high-sensitivity electrochemical applications.

**Figure 7:**
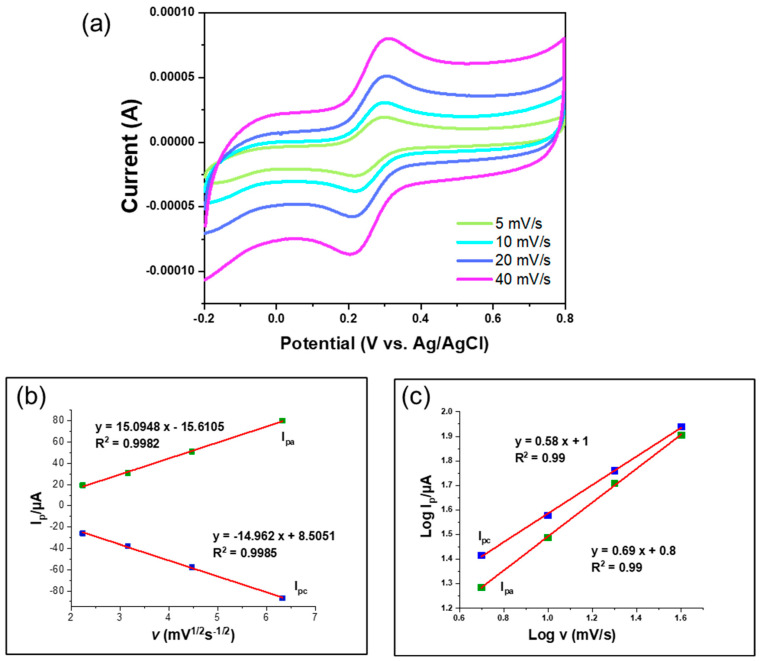
(**a**) Cyclic voltammetry plots of CeO_2_/rGO/GCE (in a 1:1 molar ratio) with increased scan rate (5, 10, 20, 40 mV/s) in the presence of 1 mM K_3_[Fe(CN)_6_] in 0.5 M KCl. (**b**) Redox peak current as a function of the square root of the scan rate with a linear fitting. (**c**) The linear relationship between the logarithm of the redox peak current and the logarithm of the scan rate.

Figure 7 illustrates the cyclic voltammetry responses of the CeO_2_/rGO/GCE composite electrode at various scan rates (5, 10, 20, and 40 mV/s). The CV curves show that the peak currents for both anodic and cathodic processes increased with the scan rate. The linear relationship between the peak current and the square root of the scan rate (Figure 7b) suggests diffusion-controlled behavior, with high correlation coefficients (R^2^ = 0.9982 for I_pc_ and R^2^ = 0.9985 for I_pa_), indicating that the CeO_2_/rGO/GCE composite electrode exhibited both surface-controlled and diffusion-controlled behavior, with effective electron transfer and high electrochemical activity. Additionally, the linear relationship between the logarithm of the redox peak current and the logarithm of scan rate (Figure 7c) was calculated with a slope of 0.69 (I_pa_) and 0.58 (I_pc_), which is close to 0.5, indicating that the diffusion-controlled mechanism dominated the reaction.

**Figure 8:**
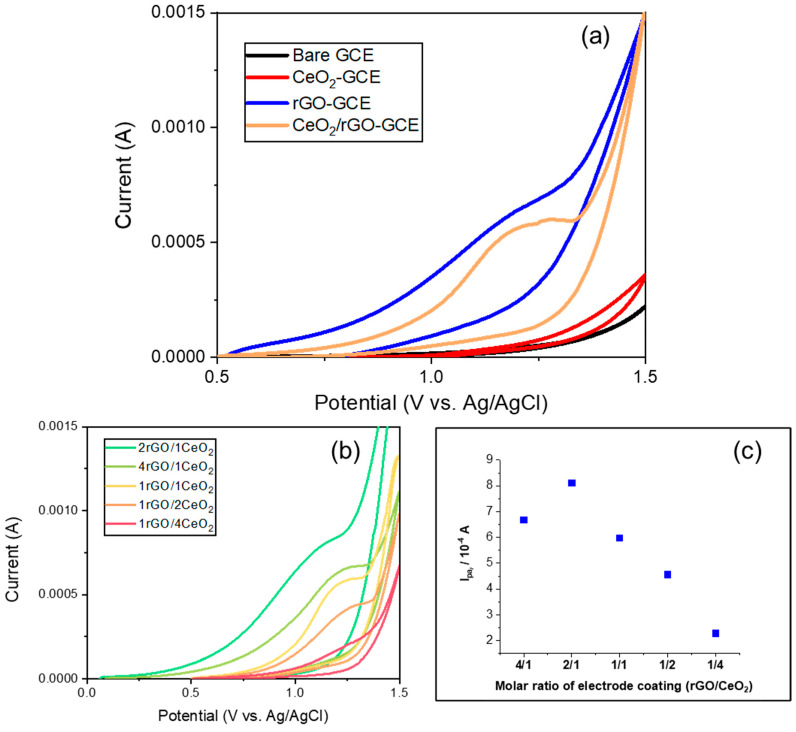
(**a**) Cyclic voltammetry plots of GCE with different coatings (bare GCE, CeO_2_/GCE, rGO/GCE, CeO_2_/rGO/GCE) in the presence of 1 M NH^4+^ in 0.5 M KCl, with a scan rate of 20 mV/s; (**b**) CV plots of CeO_2_/rGO/GCE with different molar ratios (rGO/CeO_2_ = 4:1, 2:1, 1:1, 1:2, 1:4) in the presence of 1 M NH^4+^ in 0.5 M KCl and a scan rate of 20 mV/s; (**c**) anodic peak current (I_pa_) value as a function of the composite ratio.

To analyze the sensitivity of NH^4+^, Figure 8 illustrates the CV plots of GCE with different coatings and various CeO_2_/rGO ratios. Figure 8a shows CV plots of the bare GCE, CeO_2_/GCE, rGO/GCE, and CeO_2_/rGO/GCE (molar ratio 1:1) in 0.5 M KCl with 1 M NH_4_⁺ at a scan rate of 20 mV/s. The bare GCE and CeO_2_/GCE electrodes exhibited a negligible current response, indicating they were not directly involved in the oxidation reactions. The rGO/GCE demonstrated a much higher current response due to rGO’s high electrical conductivity and large surface area, which facilitated better interaction with the NH_4_⁺ ions. However, the rGO/GCE showed an unclear peak, indicating a low selectivity. The CeO_2_/rGO/GCE kept the high current response with a higher selectivity, which was attributed to the synergistic effect between CeO_2_ and rGO; the mechanism of the reaction between the NH^4+^ ions and working electrodes is shown in Formula (3), where CeO_2_ has a unique oxygen vacancy structure that can transform between Ce^4+^ and Ce^3+^, thus acting as a bridge for the oxidation process of NH^4+^ (Formula (4)):

(3) $${NH}_{4}^{+}+rGO\;\to {NH}_{3}+rGO+e^{-}$$

(4) $${{Ce}^{4+}+e}^{-}\;\to \;{Ce}^{3+}$$

To study the current response effect on the composite ratio, Figure 8b presents CV plots of the GCE coated with different CeO_2_/rGO ratios (4:1, 2:1, 1:1, 1:2, 1:4) under the same conditions. The 2rGO/1CeO_2_ ratio exhibited the highest current response (0.82 mA, Figure 8c), indicating the most effective combination for enhancing electrochemical activity and sensitivity to NH_4_⁺, and much higher than the pure rGO coating (0.64 mA, Figure 8a). The 1rGO/2CeO_2_ and 1rGO/4CeO_2_ ratios exhibited moderate current responses, suggesting that a higher CeO_2_ content may hinder performance due to potential agglomeration and reduce the sensitivity.

**Figure 9:**
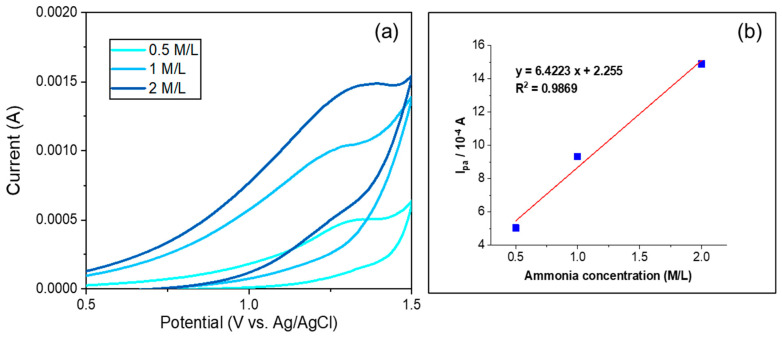
(**a**) Cyclic voltammetry plots of CeO_2_/rGO/GCE (in a 1:1 molar ratio) in the presence of increased ammonia concentrations (0.5, 1, 2 M/L) in a 0.5 M KCl solution. (**b**) Anodic peak current (I_pa_) as a function of the ammonia concentration with a linear fitting.

The CV plots of CeO_2_/rGO/GCE (in a 1:1 molar ratio) in the presence of increased ammonia concentrations (0.5, 1, and 2 M/L) in 0.5 M KCl solution are shown in Figure 9a. The current response significantly increased, where the increased current response suggests that the electrode was highly sensitive to changes in the ammonia concentration. The corresponding anodic peak current (I_pa_) as a function of the ammonia concentration with a linear fitting is shown in Figure 9b, with the data points exhibiting a clear linear trend, as confirmed by the linear fitting (R^2^ = 0.9869). This linear relationship demonstrated that the peak current increased proportionally with the ammonia concentration, which allowed for accurate quantification based on the measured current. The enhanced current response and linearity highlight the effectiveness of the CeO_2_/rGO/GCE electrode for ammonia detection.

## 4. Conclusions

In this research, we explored the focused irradiation light on the preparation of CeO_2_ nanoparticles (NPs) and investigated the enhanced properties and performance of a CeO_2_/rGO coating on GCE electrodes. The increased irradiation time initially decreased the particle size due to efficient ablation and rapid cooling, but prolonged times led to aggregation due to higher nanoparticle concentrations. Higher fluence levels resulted in smaller primary particles but also induced significant melting and aggregation, which caused secondary particles to increase in size. UV–vis spectroscopy confirmed that the high production of CeO_2_ nanoparticles by focused light was significantly higher than non-focused irradiation processes, especially at a 4 J/cm^2^ fluence. The CeO_2_/rGO composites were shown to have uniformly dispersed CeO_2_ NPs between the rGO sheets, which enhanced the synergistic effect and provided high conductivity, as confirmed by SEM, EDS mapping, and XRD analysis. The cyclic voltammetry data demonstrated that the CeO_2_/rGO composite electrodes exhibited superior electrochemical activity compared with the single-component electrodes, where the 2rGO/1CeO_2_ ratio showed the highest current response and sensitivity. The CV response to varying ammonia concentrations exhibited a linear relationship, indicating the electrode’s capability for accurate quantification. Overall, this study highlighted the successful synthesis of uniformly dispersed CeO_2_ NPs with enhanced production efficiency and the development of CeO_2_/rGO composites with superior electrochemical properties for potential applications in ammonia sensing.

## Data Availability

The data presented in this study is contained within this article.

## Supplementary Materials

The following supporting information can be downloaded at: https://www.mdpi.com/article/10.3390/nano14151238/s1, Figure S1. Absorption peak intensity as a function of CeO_2_ nanoparticle concentration and the linear fitting.
