# Supplementary material for: Optimized Ammonia-Sensing Electrode with CeO2/rGO Nano-Composite Coating Synthesized by Focused Laser Ablation in Liquid
Source: Nanomaterials (Basel). 2024 Jul 23;14(15):1238. doi: 10.3390/nano14151238 (PMC11314089; doi:10.3390/nano14151238)
Supplement: Supplementary file 1 [file nanomaterials-14-01238-s001.zip › nanomaterials-3106745-supplementary.pdf]

nanomaterials

Supplement information

Article

Optimized Ammonia-Sensing Electrode with CeO_2/rGO Nano-Composite Coating Synthesized by Focused Laser Ablation in Liquid

Mengqi Shi and Hiroyuki Wada *

School of Materials and Chemical Technology, Tokyo Institute of Technology, 4259 Nagatsuta-cho, Midori-ku, Yokohama 226-8502 Japan; shi.m.aa@m.titech.ac.jp

* Correspondence: wada.h.ac@m.titech.ac.jp; Postal address: 4259 Nagatsuta, #J2-41, Midori-ku, Yokohama 226-8502 Japan; Phone/Fax: +81 45 924 5362

To determine the concentration of CeO_2 nanoparticles using UV-Vis spectroscopy, a calibration curve was first established with known concentrations of CeO_2 nanoparticles (Figure S1). The UV-Vis absorbance values for these known concentrations were plotted, and a linear fit was applied to the data, resulting in the calibration equation: A=1425c−1.0125. This equation was derived from the slope (ϵl) and intercept (y_0) obtained from the linear fit, as shown in Figure S1. To calculate the concentration of unknown CeO_2 suspensions, the following steps were performed: (1) Measure the absorbance (A) of the CeO_2 suspension at a specific wavelength using a UV-Vis spectrophotometer (Figure 4). (2) Apply the calibration equation: c=1425A+1.0125 to solve for the unknown concentration (c). This method ensures accurate quantification of CeO_2 nanoparticle concentrations based on their absorbance values.

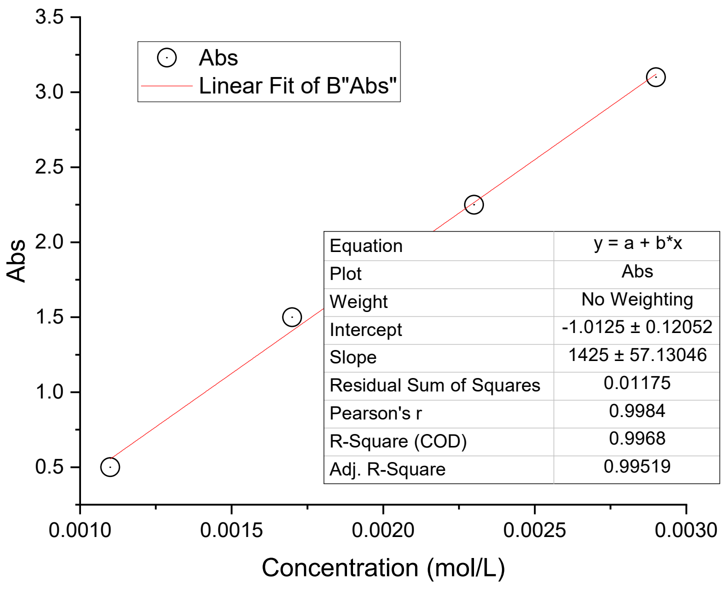

Figure S1. Absorption peak intensity as a function of CeO_2 nanoparticle concentration and the linear fitting.
